# Perceptual Learning With Tactile Stimuli in Rats: Changes in the Processing of a Dimension

**DOI:** 10.1037/xan0000104

**Published:** 2016-07

**Authors:** Luke M. Montuori, R. C. Honey

**Affiliations:** 1 School of Psychology, Cardiff University

**Keywords:** discrimination, generalization, latent inhibition, dimension, touch

## Abstract

Four experiments with male rats investigated perceptual learning involving a tactile dimension (A, B, C, D, E), where A denotes 1 end of the continuum (e.g., a rough floor) and E the other (e.g., a smooth floor). In Experiment 1, rats given preexposure to A and E learned an appetitive discrimination between them more readily than those not given preexposure. Experiment 2a showed that rats preexposed to B and D acquired a discrimination between A and E more readily than those preexposed to A and E; and in Experiment 2b the same preexposure treatments had no effect on the acquisition of a discrimination between B and D. In Experiments 3a and 3b, rats given preexposure to C learned a discrimination between A and E more readily than those not given preexposure. Experiment 4 demonstrated that preexposure to a texture (e.g., B) that was adjacent to the to-be-discriminated textures (e.g., C and E) facilitated a discrimination between them relative to preexposure to their midpoint (D). These novel perceptual learning effects are interpreted as reflecting a redistribution of processing between the notional elements of the texture dimension.

In their evocative accounts of perceptual learning, Gibson (1967) and James (1890) drew on everyday examples where experience was assumed to have affected the sense of touch. While Gibson pointed to the feats of skilled wool graders, James described how professional traders could “recognize, by feeling the flour in a barrel, whether the wheat was grown in Iowa or Tennessee” (p. 509). Demonstrations of perceptual learning involving tactile discriminations now abound in humans (e.g., Sathian & Zangaladze, 1998; see also, Rodríguez & Angulo, 2014), but this is not the case in nonhuman animals, where studies have tended to use visual stimuli or flavors. Thus, early research showed that rats were better able to learn a reinforced discrimination involving previously exposed (preexposed) visual stimuli than novel stimuli (e.g., Gibson & Walk, 1956; Hall, 1980, 1991); whereas more recent investigations began with the finding that the generalization of an aversion between one flavor and another was less marked when rats had received preexposure to both flavors than when both were novel (e.g., Honey & Hall, 1989; Mackintosh, Kaye, & Bennett, 1991; Symonds & Hall, 1995). While there are notable examples where the key observations from studies of perceptual learning in rats find counterparts in birds and people (e.g., Honey, Bateson, & Horn, 1994; Mitchell & Hall, 2014; Montuori & Honey, 2015), one cannot simply assume that all sensory domains will be shaped in the same way by experience. Also, theoretical analyses that have been developed in the context of some classes of stimuli (e.g., flavor “cocktails” and geometric forms) might not be applicable to others. These considerations led us to embark on the current series of experiments, which investigated perceptual learning with tactile stimuli in rats.

Extensive analysis of the systems that underlie the sense of touch in rodents, specifically those involving their whisker system, have provided detailed information about both the requisite neural circuits (Brecht, 2007; Carvell & Simons, 1990; Diamond, von Heimendahl, Knutsen, Kleinfeld, & Ahissar, 2008) and plasticity in the associated (barrel) cortex (Fox, 2002, 2008; see also, Oswald et al., 2001; Ramos, 2014). Given this interest it is perhaps surprising that behavioral analysis of perceptual learning with tactile stimuli in rats has been very limited (see Diamond et al., 2008). Indeed, the few studies that have employed tactile stimuli were not designed to demonstrate that preexposure to such stimuli results in perceptual learning effects of the sort described by Gibson (1967) and James (1890). However, they do help to illustrate both the effect of interest and one simple theoretical analysis of perceptual learning.

Experiments involving maze learning in rats have demonstrated that preexposure to different floor coverings in the arms of a radial maze (red sandpaper and black rubber), can facilitate the acquisition of a discrimination where food is later made available at the end of the arm with one floor covering but not at the end of the arm with the other floor covering (Chamizo & Mackintosh, 1989). This perceptual learning effect was interpreted as reflecting a redistribution of processing between the unique and common elements of the exposed arms. To take the concrete example provided by Chamizo and Mackintosh (1989): If the different floor types are considered the unique elements of the two arms (denoted A and B) and the shared visual characteristics of the arms are their common elements (denoted X), then preexposure to AX and BX will result in a greater reduction in the processing of X than of A and B. Most obviously, because X is exposed on twice as many occasions as either A or B. Under these conditions, the unique elements (A and B) will be better placed to enter into association with the presence or absence of food (Lubow, 1973) during discrimination learning than will the irrelevant, common elements (see Honey & Hall, 1989; McLaren, Kaye, & Mackintosh, 1989).

Although the specific analysis outlined above seems to be inconsistent with the observation that rats in the control group were exposed to X alone (a maze arm with a green plastic floor; see p. 24, Chamizo & Mackintosh, 1989) subsequent experiments lent support to it. For example, in one experiment the walls of the arm with the red sandpaper floor were painted black, while those of the arm with the black rubber floor were painted white. Under these conditions, preexposure to the arms retarded later discrimination learning relative to a group that was simply placed in the radial maze. In this case, limiting the degree of overlap between the visual stimuli in the two arms meant that preexposure simply served to reduce the processing of those unique visual stimuli (or potentially the textures) that defined the to-be-discriminated arms (see Chamizo & Mackintosh, 1989; Trobalon, Sansa, Chamizo & Mackintosh, 1991). Perhaps more telling is the fact that a discrimination in which the floor coverings (A and B) were relevant, and the extramaze cues were irrelevant to whether food would be available at the end of two arms, was facilitated by preexposure to the extramaze cues (X) alone (see Experiment 1B, Trobalon, Chamizo, & Mackintosh, 1992). Leaving to one side the fact that differences between the tactile stimuli (rough and smooth) were correlated with differences in their visual characteristics (red and black), the latter results do not require that preexposure to the tactile stimuli (the floors) affected their processing or discriminability. They were not intended to do so. Rather, they only require that the processing of the extramaze cues had changed during the preexposure stage.

The results outlined above confirm that rats can learn discriminations involving floor types (see Lawrence, 1949; see also Oswald et al., 2001; Ramos, 2014). This observation, coupled with the plasticity of the requisite cortical mechanisms (Fox, 2002, 2008), is consistent with the idea that perceptual learning effects might be observed in rats given preexposure to floor-dwelling tactile stimuli. Our point of departure in Experiment 1 was to examine the effect of preexposure to two such tactile stimuli (henceforth textures), that covered the floor of a standard operant chamber, on the acquisition of an appetitive discrimination involving the textures. These textures were created using different grades of sandpaper. Having demonstrated a perceptual learning effect in Experiment 1, Experiments 2–4 examined the origin of this effect by making use of the fact that a texture dimension (rough to smooth) can be conveniently generated using commercially available grades of sandpaper.

## Experiment 1

The design of Experiment 1 is summarized in Table 1 and involved two stages: preexposure and discrimination. In both stages, rats were placed in a standard operant chamber with modifications that enabled the floor to be covered with sandpaper. There were two groups that received different treatments during the preexposure stage. For Group Control, there was no sandpaper on the floor during the preexposure stage, and the rats in this group were simply placed in an operant chamber with a flat aluminum floor. For Group Preexposed, the flat aluminum floor was covered, on different trials, with one of two types of sandpapers (A and E). Thus, both groups received equivalent preexposure to the features of the operant chamber other than the floor textures, and any perceptual learning effect is therefore unlikely to be a consequence of differential preexposure to these features (cf. Chamizo & Mackintosh, 1989). During the discrimination stage, all rats entered the same chambers where the presence of floor A was paired with the delivery of food, whereas the presence of E in the same chamber was not. Discrimination learning was monitored by recording food well activity during the food-free periods at the start of each trial.[Table-anchor tbl1]

### Method

#### Subjects

Sixteen naïve male Lister hooded rats (*Rattus norvegicus*; supplied by Harlan Olac, Ltd., United Kingdom) served in Experiment 1. They were approximately 3 months old at the start of the experiment and were maintained at between 80% and 85% of their ad libitum weights by being given a restricted amount of food at the end of each day. They were housed in pairs in a climate-controlled room with a 12-hr light/dark cycle and given free access to water. All experimental procedures were conducted during the light part of the cycle.

#### Apparatus

Four operant chambers (Campden Instruments, Ltd., Loughborough, United Kingdom: Test Chamber CI-410), arranged in a 2 × 2 grid, were used. Each was contained within a sound-attenuating shell, the door to which remained open during the experiment. Each chamber (24.5 cm × 23 cm × 21 cm; W × D × H) had three aluminum walls, an aluminum ceiling and an aluminum floor that could be covered with sandpaper. The front wall of the chamber was made from transparent Perspex. This wall served as the door and allowed ambient illumination from the experimental room to enter the chamber; in later experiments illumination of the chambers was limited by extinguishing these room lights. There was a food well in the left-hand wall (5 cm × 4 cm × 6 cm: W × D × H) into which 45-mg TestDiet food pellets (supplied by MLab, Richmond, IN) could be delivered. A top-hinged transparent plastic flap guarded access to this food well. When this flap was moved by approximately 2 mm, a food well entry response was recorded. Two grades of sandpaper (Wickes, United Kingdom) were used, with the average grit size specified by ISO designations p40 and p100 (grit sizes 425 μm and 162 μm, respectively). Two of the edges to the sandpaper extended beyond both the back wall of the chamber and the door, which made them inaccessible to the rats. The edges that remained within the chambers were protected with PVC A4 slide binders; and the front edge of the sandpaper that extended beyond the door was secured to the aluminum floor with a bulldog clip.

#### Procedure

There were two principal stages: preexposure and discrimination training. On each of the four preexposure days, rats were placed in the operant chambers for four separate 3-min periods that were separated by an interval of, approximately, 1 min. During this 1-min period they were removed from the chamber and placed in a holding cage. For Group Control, the floor of the chamber was aluminum, and for Group Preexposed this floor was covered with the designated grade of sandpaper (A or E). Half of the rats in Group Preexposed received A, E, E, A on each day while the remainder received E, A, A, E on each day. For half of the rats in both groups, A was the rough stimulus (i.e., p40) and E was smooth (i.e., p100) and for the rest this arrangement was reversed.

Rats were then trained to retrieve food pellets from the food well in a 20-min session on each of 2 days. On both days, food pellets were delivered on a variable time 60-s schedule. On Day 1, the flap in front of the food well was fixed in a deflected position, rendering the food well accessible, and on Day 2 the flap was returned to its vertical position and rats needed to displace it in order to gain access to the food pellets. On each of the following 6 days of discrimination training there were four 5-min sessions (two reinforced and two nonreinforced) that were separated by, approximately, 60 s. During reinforced sessions, two food pellets were delivered on a fixed-time 30-s schedule, and during nonreinforced trials no food was presented. The sequence according to which the reinforced and nonreinforced trials were delivered was counterbalanced within a day: food, no food, no food, food for half of the rats, and no food, food, food, no food, for the remainder. These sequences alternated across days. The frequency of food well entries was recorded during the first 30 s of each session when no food was delivered in either type of session, and a discrimination ratio derived: rate of responding during reinforced trials divided by the rate of responding during reinforced and nonreinforced trials. Using this ratio, scores above .50 indicate that the rate of responding on reinforced trials is higher than on nonreinforced trials.

### Results and Discussion

**Figure 1 fig1:**
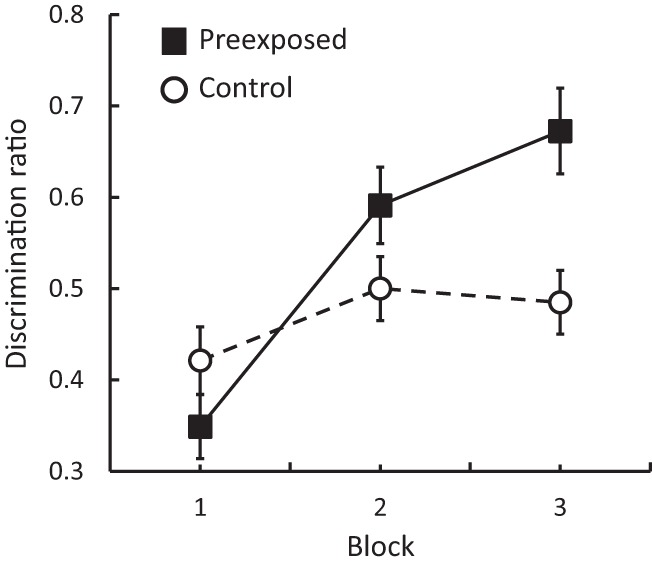
Experiment 1: Mean discrimination ratios (±SEM) in Groups Preexposed and Control.

The results from the discrimination training stage in Experiment 1 are presented in Figure 1 in 2-day blocks. Inspection of this figure shows that initially the discrimination ratios were below .50 in both groups. This was a consistent observation across the set of experiments described here, and is most simply explained by the nature of the constrained sequences that were used within a day of discrimination training: food, no food, no food, food; or no food, food, food, no food. Within these sequences, carryover of responding from one trial to the next will oppose the anticipated pattern of responding (food well entries on food trials, but not on no food trials) on two of the three transitions within a day. Examination of Figure 1 also shows that as discrimination training proceeded performance improved in Group Preexposed, but this improvement was not evident in Group Control, and by the final block there was a marked difference between the two groups. This description was supported by the results of an ANOVA that revealed no significant effect of group, *F*(1, 14) = 2.79, *p* > .11, a significant effect of block, *F*(2, 28) = 20.53, *p* < .001, η_p_^2^ = .59, and an interaction between these factors, *F*(2, 28) = 8.22, *p* < .005, η_p_^2^ .37. Analysis of simple main effects confirmed that there was an effect of block in Group Preexposed, *F*(2, 14) = 18.62, *p* < .001, η_p_^2^ = .73, but not in Group Control, *F*(2, 14) = 3.06, *p* > .07; and there was a difference between the groups on Block 3, *F*(1, 14) = 10.01, *p* < .01, η_p_^2^ = .42, 95% CI [.08, .61], but not on the other blocks, largest *F*(1, 14) = 2.72, *p* > .12. A one-sample *t* test confirmed that the scores for Group Preexposed were above 0.50 on this final block, *t*(7) = 3.63, *p* < .01. The overall rate of food well entries, with means of 22.80 rpm for Group Control and 21.65 rpm for Group Preexposed, did not differ significantly (*F* < 1), and this fact suggests that the introduction of a novel floor covering in Group Control did not result in a general disruption to performance (i.e., to food well entries). The pattern of results are instead consistent with the view that the perceptual learning effect observed in Experiment 1 has a different origin, based on a change in the processing of the tactile stimuli. To reduce the possibility that any effects were based on changes in the processing of their visual characteristics, later experiments were conducted with the experimental rooms extinguished.[Fig-anchor fig1]

## Experiments 2a and 2b

One simple interpretation for the results of Experiment 1 follows from the observation that preexposure to two similar stimuli will mean that the elements that they have in common will have been exposed more frequently than their unique elements. In the case of the textures used in Experiment 1, we can denote the more frequently presented common elements as X and the less frequently presented unique elements as A and E, and make the simplifying assumption for now that all of the stimuli between A and E will also activate X. If preexposure to A and E results in a greater reduction in the processing of X than of A and E, then discrimination learning should be improved relative to group control for which A, E, and X are more equally processed at the outset of discrimination training. This analysis immediately suggests conditions that should result in a more marked perceptual learning effect than preexposure to A and E. Thus, if rats received preexposure to B and D then this should affect a reduction in the processing of the common element X, but it should be less likely to result in a reduction in the processing of the unique elements of A and E than would preexposure to A and E. On these grounds, preexposure to B and D should result in a more marked perceptual learning effect than preexposure to A and E when the test discrimination involves A and E. This prediction was assessed in Experiment 2a in Groups AE/AE versus BD/AE (see Table 1); with the letters before the oblique lines denoting the preexposed stimuli and those after the line denoting the stimuli presented during discrimination training.

In Experiment 2b, we contrasted the effect of these two forms of preexposure (to A and E and to B and D) on the acquisition of a discrimination between B and D. Application of the same form of analysis to this comparison would, without further assumptions, yield the complementary prediction: The discrimination between B and D should proceed more readily after preexposure to A and E than after preexposure to B and D, because both would affect a reduction in the processing of X, but preexposure to B and D would also result in a reduction in the processing of the (unique) elements that are relevant to the tested discrimination. However, a moment’s reflection reveals that this prediction ignores the obvious possibility that stimuli that are closer together on the texture dimension, if that is how it should be treated, will share a greater proportion of common elements, and be more difficult to discriminate. While preexposure to A and E might be less likely to impact on the elements that are uniquely activated by B and D than would preexposure to B and D, preexposure to B and D might result in a more complete reduction in the processing of their common elements than would preexposure to A and E. In this case then, the effect of the different types of preexposure (A and E or B and D) will be determined by the relative contribution of these two opposing effects. The value of such additional considerations will be evident should the discrimination involving B and D proceeds less readily than that involving A and E, as would be expected if the way that we have characterized our stimulus set (as an ordered dimension from A through B and C to D to E) reflects how the rats process the different floor types.

It is worth noting, at this juncture, that an analogous comparison was made by Scahill and Mackintosh (2004) using a flavor-aversion procedure in rats (see also Suret & McLaren, 2003). They found no evidence that preexposure to two easily distinguishable flavors (AX and BX or A and B) was any more effective than preexposure to two hard to discriminate flavors (aX and bX; where the intensity of the unique elements was reduced) in allowing rats to learn a discrimination in which aX was paired with illness and bX was not (but see, Sanjuán, Nelson, & Alonso, 2014).

### Method

Thirty-two naïve male Lister hooded rats (*Rattus norvegicus*) served in Experiments 2a (*n* = 16) and 2b (*n* = 16). The rats were, approximately, 3 months old at the start of the experiment and were housed and maintained in the same way as Experiment 1. The apparatus and procedure were the same as in Experiment 1 with the exceptions that the four grades of sandpaper that served as A, B, D, and E were: p60, p80, p150, p320 (grit sizes 269 μm, 201 μm, 100 μm, 46.2 μm, respectively; 3M, United Kingdom). In order to reduce the likelihood that differences in the visual features of the sandpapers were being used by rats to discriminate between them, there was no local illumination within the chambers, and the lights in the experimental room were now turned off. There was very limited illumination from (a) the computer screen that was located within the experimental room, and (b) the corridor outside of the experimental room, that entered the room from around the doorframe. In the same way as in Experiment 1, on each of the four preexposure days, all rats were placed in the operant chambers for four separate 3-min periods that were separated by an interval of, approximately, 1 min. In Experiment 2a, half of the rats received preexposure to A and E (p60 and p320), and the remainder received preexposure to B and D (p80 and p150). After the rats had been trained to retrieve food pellets from the food well, there were 6 days of discrimination training involving A and E that proceeded in the same way as in Experiment 1. The two groups are designated AE/AE and BD/AE to indicate the stimuli presented during the two key stages: preexposure/discrimination. In Experiment 2b, again half of the rats received preexposure to A and E and half to B and D; and after these rats had been trained to retrieve food pellets from the food well, there were 6 days of discrimination training involving B and D. These two groups are designated AE/BD and BD/BD. For half of the rats in each of the groups in Experiments 2a and 2b, the rougher stimulus was reinforced and the smoother stimulus was not, and for the remaining half the opposite was true. Details of the procedure that have not been mentioned were the same as in Experiment 1.

### Results and Discussion

**Figure 2 fig2:**
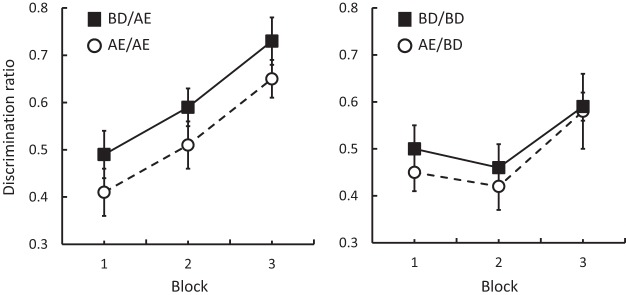
Experiment 2: Mean discrimination ratios (±SEM) in Groups AE/AE and BD/AE (Experiment 2a; left panel); and Groups AE/BD and BD/BD (Experiment 2b; right panel). The letters before the oblique (/) indicate the stimuli that were preexposed and those after the oblique indicate the to-be-discriminated stimuli.

Figure 2 depicts the results from Experiments 2a (left panel) and 2b (right panel). Comparison of the panels suggests that the discrimination between A and E (left panel) was acquired more rapidly than that between B and D (right panel). It is also apparent from the left panel that the discrimination between A and E developed more readily after preexposure to B and D (i.e., in Group BD/AE) than after preexposure to A and E (i.e., in Group AE/AE). ANOVA conducted on the results from Experiment 2a confirmed that there was an effect of group, *F*(1, 14) = 4.90, *p* < .05, η_p_^2^ = .26, 95% CI [.004, .49], and block, *F*(2, 28) = 13.33, *p* < .001, η_p_^2^ = .49, but there was no interaction between these factors, *F* < 1. In contrast, acquisition of the discrimination between B and D in Experiment 2b was not affected by these two forms of preexposure (i.e., in Groups BD/BD and AE/BD; cf. Scahill & Mackintosh, 2004; Suret & McLaren, 2003; but see Sanjuán et al., 2014). ANOVA revealed an effect of block, *F*(2, 28) = 3.87, *p* < .05, η_p_^2^ = .22, but no effect of group and no interaction between these factors, *F*s < 1. The main effect of block reflected the fact that the scores on Block 1 differed from Block 3, *F*(1, 15) = 4.33, *p* = .05, η_p_^2^ = .22, and the scores on Block 2 differed from Block 3, *F*(1, 15) = 6.98, *p* < .05, η_p_^2^ = .32. An analysis of the overall rates of magazine entries demonstrated no difference in these rates between the preexposure conditions in either experiment, with means of 11.27 rpm for Group AE/AE and 11.78 rpm for Group BD/AE in Experiment 2a, *F* < 1; and 7.48 rpm for Group AE/BD and 7.51 rpm for Group BD/BD in Experiment 2b, *F* < 1.[Fig-anchor fig2]

The general observation that the discrimination between A and E proceeded more readily than that between B and D is consistent with our treatment of the stimuli as forming a dimension where the proximity of the designated letters in the alphabet relates to their similarity to the rat. The fact that the discrimination involving A and E was acquired more rapidly after preexposure to B and D than after preexposure to A and E receives a ready explanation in terms of the redistribution of processing between the elements of A and E: Preexposure to B and D will reduce the effective processing of the elements that are common to A and E (i.e., X), but will leave their unique elements (A and E) more effective than will preexposure to A and E. As we have already noted, once it is allowed that B and D share elements that A and E do not, there is no good reason to predict that the two forms of preexposure (to A and E or to B and D) will have different effects on the acquisition of a discrimination is between B and D.

## Experiment 3

The most direct prediction that follows from the analysis of the patterns of results observed in Experiments 1 and 2 is that preexposure to a single texture (C) that lies between to-be-discriminated stimuli (e.g., A and E) should be sufficient to generate a perceptual learning effect (cf. Mundy, Honey, & Dwyer, 2007). This prediction was tested in Experiments 3a and 3b in which rats in Group Midpoint were preexposed to the notional midpoint (C) while those in Group Control were simply placed in the apparatus, and then both groups received a discrimination between A and E (see Table 1). The two experiments were similar, with the exception that in Experiment 3a rats in Group Control were placed on the metal floor during the preexposure stage, whereas in Experiment 3b they were placed on the smooth paper side of the sandpaper.

### Method

Sixteen naïve male Lister hooded rats (*Rattus norvegicus*) served in both Experiments 3a and 3b. They were, approximately, 3 months old at the start of the experiment and their housing and maintenance was the same as in Experiment 1. The apparatus and procedure were the same as in Experiments 1 and 2 with the exceptions that rats in Group Midpoint, were preexposed to the chamber with the floor covered by a grade of sandpaper (C: p80, 201mμ, Experiment 3a; and p100, 162 μm, Experiment 3b) that was located between the to-be-discriminated stimuli (A and E: p40 and p100 = 425 μm and 162 μm, respectively, Experiment 3a; and p80 and p150 = 201 μm and 100 μm, respectively, Experiment 3b); and those in Group Control were simply placed in the chamber with either a metal floor (Experiment 3a) or the smooth, paper side of a sheet of sandpaper (Experiment 3b). The to-be-discriminated stimuli in Experiment 3a were the same as Experiment 1, and those used in Experiment 3b were the same as Experiment 2b.

### Results

**Figure 3 fig3:**
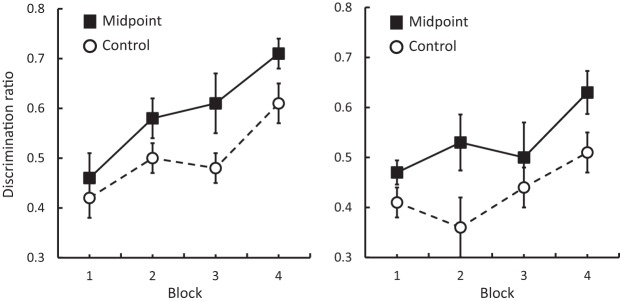
Experiment 3: Mean discrimination ratios (±SEM) in Groups Midpoint and Control from Experiments 3a (left panel) and 3b (right panel).

The results from Experiments 3a and 3b are presented in the left and right panels of Figure 3, respectively. Examination of both panels shows that as training progressed discrimination performance improved, and that this improvement was more rapid in Group Midpoint than in Group Control. As in Experiment 2b, the discrimination between the p80 and p150 sandpapers in Experiment 3b proved to be difficult, but in this case acquisition of the discrimination was affected by the preexposure stage. ANOVA conducted on the results from Experiment 3a confirmed that there was an effect of group, *F*(1, 14) = 5.84, *p* < .05, η_p_^2^ = .29, 95% CI [.02, .52], and block, *F*(3, 42) = 10.61, *p* < .001, η_p_^2^ = .43, but no interaction between these factors, *F* < 1. The overall levels of food well entries in Experiment 3a, with means of 22.81 rpm for Group Control and 24.89 rpm for Group Midpoint did not differ significantly, *F* < 1. A parallel ANOVA conducted on the results from Experiment 3b confirmed that there was an effect of group, *F*(5, 14) = 5.37, *p* < .05, η_p_^2^ = .28, 95% CI [.01, .51], and block, *F*(3, 42) = 3.94, *p* < .05, η_p_^2^ = .22, and no interaction between these factors, *F* < 1. The overall levels of food-well entries, with means of 24.95 rpm for Group Control and 27.55 rpm for Group Midpoint did not differ significantly, *F* < 1. By the final block of training, 15 of the 16 rats who received midpoint preexposure in Experiments 3a and 3b had discrimination ratios that were above .50.[Fig-anchor fig3]

### Discussion

The results of Experiment 3 confirm that a perceptual learning effect can be observed by simply exposing rats to the midpoint between the to-be-discriminated textures. More specifically, Experiment 3 shows that preexposure to a midpoint texture can improve later discrimination learning relative to a control condition (preexposure to the smooth paper side of a sheet of sandpaper) when both treatments have taken place in the dark (cf. Experiment 1). This observation is consistent with the analysis of the results of Experiment 2, where preexposure to two textures (B and D) was more effective in improving later discrimination involving A and E than was preexposure to A and E. The results also provide a replication of an effect originally reported by Mundy et al. (2007) where preexposure to the midpoint on a morph between two similar faces was sufficient to produce a perceptual learning effect in humans. In Experiment 4 we explore a novel prediction that can be derived from our analysis of the results of Experiments 1–3 that has not been evaluated in previous studies of perceptual learning.

## Experiment 4

There has already been cause to argue that stimuli closer together on our presumed texture dimension (e.g., A and C) are more similar to one another than stimuli that are further apart (e.g., A and E; see Experiments 2a and 2b). This argument has a further implication in the context of preexposure to the midpoint. Thus, preexposure to a stimulus, B, that is the midpoint of A and C, will result in a reduction in the efficacy of the common X elements that A shares with C, but B will also have some elements that it shares exclusively with A and others that it shares exclusively with C; these elements are subsets of those uniquely activated by A and C. Preexposure to the midpoint, B, will thus reduce the effectiveness of both the elements that are common to two stimuli (in this case A and C), but it could still have some impact on their unique elements. Based on this observation, preexposure to a stimulus that is adjacent to the to-be-discriminated stimuli (e.g., D) might allow a discrimination between A and C to proceed more readily than preexposure to the midpoint. Thus, preexposure to D will result in a reduction in the efficacy of some of the elements that A shares with C (i.e., X) and to elements that D shares specifically with C, whereas preexposure to B will result in a reduction in the efficacy of X and elements that B shares with A and others that B shares with C. On this basis, the discrimination between A and C might be expected to proceed more readily after preexposure to D than after preexposure to B; and similarly the discrimination between C and E should proceed more readily after preexposure to B than after preexposure to D. Experiment 4 tested this prediction in two groups. The midpoint group received either (a) preexposure to B and then a discrimination between A and C, or (b) preexposure to D and then a discrimination between C and E. The adjacent group received either (a) preexposure to D and then a discrimination between A and C, or (b) preexposure to B and then a discrimination between C and E (see Table 1). With this design, a given texture (e.g., B) serves as the midpoint stimulus for one discrimination (e.g., A and C) and the adjacent stimulus for the other discrimination (e.g., C and E).

### Method

Sixteen naïve male Lister hooded rats (*Rattus norvegicus*) served in Experiment 4. They were approximately 3 months old at the start of the experiment and maintained in the same way as in previous experiments. The apparatus and procedure were the same as in Experiment 1 with the exception that five grades of sandpaper were required instead of two (p60, p80, p100, p150, p320; grit sizes 269 μm, 201 μm, 162 μm, 100 μm, 46.2 μm, respectively; 3M, United Kingdom). These five stimuli served as A, B, C, D, and E. Half of the rats in Group Midpoint received preexposure to B (p80) before discrimination training involving A and C (p60 and p100), and the remainder received preexposure to D (p150) before discrimination training involving C and E (p100 and p320). Half of the rats in Group Adjacent received preexposure to B (p150) before discrimination training involving C and E (p60 and p100) and the rest received preexposure to D (p60) before discrimination training involving A and C (p100 and p320). The identity of stimulus that was reinforced (A or C and C or E) was counterbalanced in both Groups Midpoint and Adjacent.

### Results and Discussion

**Figure 4 fig4:**
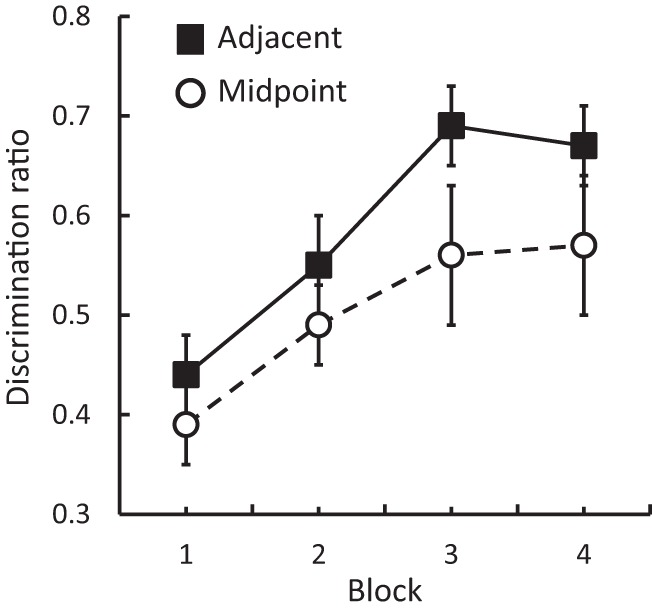
Experiment 4: Mean discrimination ratios (±SEM) in Groups Adjacent and Midpoint.

The results of Experiment 4 are presented in Figure 4. Inspection of this figure shows that there was an improvement in discrimination over the course of the four 2-day blocks of training, and that this improvement was more evident in Group Adjacent than in Group Midpoint. ANOVA with group and block as factors revealed significant effects of group, *F*(1, 14) = 6.15, *p* < .05, η_p_^2^ = .31, 95% CI [.02, .53], and block, *F*(3, 42) = 7.49, *p* < .05, η_p_^2^ = .35, and no interaction between these factors, *F* < 1. The overall levels of magazine entries, with means of 14.04 rpm for group midpoint and 17.32 rpm for group adjacent, did not differ significantly, *F*(1, 14) = 3.45, *p* > .05.[Fig-anchor fig4]

## General Discussion

Some studies of perceptual learning with rodents have included textures (floor types) as a part of the to-be-discriminated arrays (e.g., Chamizo & Mackintosh, 1989; Trobalon et al., 1991, 1992), but there has been no systematic investigation of perceptual learning with tactile stimuli per se. The paucity of such evidence contrasts with the extensive use of the rodent whisker system as a model to examine neural plasticity (e.g., Diamond et al., 2008; see also, Oswald et al., 2001; Ramos, 2014). The set of experiments described here represents the start of a behavioral analysis of the impact of experience with tactile stimuli (or textures) on their subsequent discrimination. Taken together, the results of Experiments 1–4 provide support for the view that the processing of our texture dimension changes in an orderly way as a consequence of simple preexposure, and they provide converging evidence about the nature of these changes. The results of each experiment suggest that the improvements seen in discrimination learning following stimulus preexposure reflect a redistribution of processing among the elements of the dimension, and are consistent with a variety of theoretical analyses that have assumed that changes of this kind might underpin instances of perceptual learning in other preparations and sensory domains (e.g., Hall, 2003; Honey & Bateson, 1996; McLaren et al., 1989).^1^ It should be acknowledged, however, that while the theoretical analysis of the effects of the preexposure conditions within each experiment is internally coherent, the use of different grades of sandpaper across different experiments exacerbates the difficulty of comparing their results directly. Notwithstanding this observation, the results of Experiments 1–4 provide support for the general view that the same principles might be in operation across quite different preparations (see Montuori & Honey, 2015; see also, Mitchell & Hall, 2014). Indeed some of the effects that we report have relatively direct counterparts in other preparations.

For example, the finding that preexposure to the midpoint along the texture dimension (C) facilities later discrimination learning (involving A and E; Experiment 3) is clearly compatible with an equivalent effect reported by Mundy et al. (2007) in humans using face morphs, and with the finding that preexposure to X alone is sufficient to facilitate a discrimination between the arms of a maze (AX and BX; Chamizo & Mackintosh, 1989) and between two flavor cocktails (Mackintosh et al., 1991). The observation that preexposure to two stimuli that are close to one another on the texture dimension (i.e., B and D) was more effective improving discrimination of A and E than was preexposure to A and E (Experiment 2) can be considered to be another instance of this midpoint effect. By contrast, these two forms of preexposure (to B/D and to A/E) had no effect on the acquisition of a difficult discrimination (i.e., between B and D). This observation is consistent with results from both humans (Suret & McLaren, 2003) and rats (Scahill & Mackintosh, 2004; but see Sanjuán et al., 2014).

The novel asymmetry demonstrated in Experiments 2a and 2b, between the effects of preexposure to A/E and B/D on later learning involving either A/E or B/D, can also be derived from the idea that preexposure results in a redistribution of processing among the elements of the preexposed stimuli. Thus, preexposure to A and E will be more likely to impact on the unique elements of A and E than will preexposure to B and E, with both forms of preexposure having a similar effect on the elements that A and E share. On these grounds, preexposure to B and D should result in a greater perceptual learning effect than should preexposure to A and E. In contrast, preexposure to A and E should be less likely to impact on both the common elements of B and D and their unique elements than will preexposure to B and D: these two differences should have opposing effects on later discrimination learning involving B and D. Therefore, these two forms of preexposure might not be expected to have different effects on the acquisition of a discrimination involving B and D. Finally, in Experiment 4, we showed that preexposure to a stimulus (B) that was adjacent to the to-be-discriminated stimuli (C and E) was more effective in promoting their later discrimination than was preexposure to the b midpoint (D). In this case, we argued that preexposure to B will be less likely to change the processing of those elements that C shares with E than will preexposure to D (cf. Experiment 2), but that preexposure to B will also have less impact on the unique elements of E than will preexposure to D. The fact that adjacent preexposure is more beneficial than midpoint preexposure indicates that the effect of preexposure to B (in leaving the processing of the unique elements of E high) outweighs the fact that this form of preexposure will be less effective in reducing the processing the common elements of C and E. This analysis will clearly depend on the adjacent stimulus not being too distant from the to-be-discriminated stimuli, a prediction that receives indirect support from the results of Experiment 3b in which the control treatment was exposure to a smooth paper floor.

The general idea that preexposure to exemplars from a texture dimension results in a relatively long-lasting change in the processing of that dimension leaves many issues unresolved. Most pressing perhaps is how the dimension is represented. We have fairly good evidence that our description of the stimuli as an ordered list (A, B, C, D, and E) maps onto how the rats treat the stimuli, with the most obvious being that a discrimination between exemplars that were further apart on the dimension (A and E) was acquired more readily than between exemplars that were closer together (B and D; Experiment 2). We have chosen to describe the similarity between the various exemplars in terms of the overlap between the notional elements that each is assumed to activate (Atkinson & Estes, 1963), but there are a variety of ways to implement this analysis. For example, McLaren and Mackintosh (2000; see also, Suret & McLaren, 2003) suppose that a dimension might be represented by an ordered set of units (call them 1–5), with a given stimulus activating a pattern of activity across this set (e.g., stimulus C might activate units 2–4). They assume that each unit responds most strongly to one value on a dimension and less strongly to neighboring values; and that increases in intensity are represented by a corresponding increase in the activation of the units and by the recruitment of additional, adjacent units. Within this scheme, preexposure to two stimuli (e.g., B and D) is assumed to result in a reduction in the salience of their corresponding units (1–3 and 3–5), with the result that the unit/s that are activated on each trial (in this case 3) will lose their capacity to become active at a point at which the less frequently presented (unique) elements (1, 2 and 4, 5) will have a nonzero activation value. These changes in salience will mean that a discrimination between two preexposed stimuli will occur more readily than between two novel stimuli, provided it is the case that the stimuli would tend to activate overlapping sets of units in the first instance. In the context of the present set of experiments, it is interesting to note that neurons in the barrel cortex respond preferentially to stimulation of one whisker and less to adjacent whiskers, and the adaptation in such responses following repeated stimulation can be highly whisker specific (see Katz, Heiss, & Lampl, 2006). Whether or not these neuronal processes can be tied to computational analyses of perceptual learning, and to perceptual learning at a behavioral level, remains an open issue.

The novel perceptual learning effects that we have described in Experiments 1–4 are consistent with a theoretical analysis based upon a very simple assumption: preexposure to a stimulus results in a reduction in its processing that generalizes to other similar stimuli. Analyses based upon this assumption clearly have broad explanatory power: they apply across different preparations and stimulus classes in animals (flavors, geometric patterns, spatial arrays, and now textures) and people (flavors, visual stimuli). However, such analyses will not suffice as an explanation for features of at least some instances of perceptual learning (e.g., Mundy et al., 2007). It is a matter for future research to determine whether processes beyond those considered here will be needed to provide a complete account of rodent perceptual learning involving textures.

## Figures and Tables

**Table 1 tbl1:** Design of Experiments 1–4

Group	Preexposure	Discrimination
Experiment 1		
Preexposed	A_p40_, E_p100_	A_p40_ → food, E_p100_ → no food
Control	Context alone	A_p40_ → food, E_p100_ → no food
Experiment 2a		
AE/AE	A_p60_, E_p320_	A_p60_ → food, E_p320_ → no food
BD/AE	B_p80_, D_p150_	A_p60_ → food, E_p320_ → no food
Experiment 2b		
AE/BD	A_p60_, E_p320_	B_p80_ → food, D_p150_ → no food
BD/BD	B_p80_, D_p150_	B_p80_ → food, D_p150_ → no food
Experiment 3a		
Midpoint	C_p80_	A_p40_ → food, E_p100_ → no food
Control	Context alone	A_p40_ → food, E_p100_ → no food
Experiment 3b		
Midpoint	C_p100_	A_p80_ → food, E_p150_ → no food
Control	Context alone	A_p80_ → food, E_p150_ → no food
Experiment 4		
Midpoint	B_p80_	A_p60_ → food, C_p100_ → no food
Adjacent	D_p150_	A_p60_ → food, C_p100_ → no food
Midpoint	D_p150_	C_p100_ → food, E_p320_ → no food
Adjacent	B_p80_	C_p100_ → food, E_p320_ → no food
*Note.* A, B, C, D, and E denote textures along a dimension that ranged from rough (e.g., A) to smooth (e.g., E; sandpaper grades are shown as subscripts). The textures that were followed by food or no food during discrimination training were counterbalanced.		

## Footnotes

1 Preexposure to C will disrupt the formation of an association between the elements that B and D share with one another (i.e., c) and food, and this will facilitate discrimination learning. Similarly, preexposure to C might also disrupt the formation of associations between c and the unique elements of B and D (i.e., b and d, respectively). This too would facilitate discrimination learning (see McLaren et al., 1989).
